# Genome and transcriptome analysis of *Enterococcus faecium* from intestinal colonization and *Enterococcus faecium* from urinary tract infection

**DOI:** 10.3389/fmicb.2023.1273949

**Published:** 2023-10-31

**Authors:** Ge Huang, Yizheng Zhou, Hai Cheng, Tao Lv, Lisi Zheng, Chengbin Li, Yunbo Chen

**Affiliations:** ^1^Department of Clinical Laboratory Center, Central Hospital of Enshi Tujia and Miao Autonomous Prefecture, Enshi, China; ^2^Jingzhou Hospital Affiliated to Yangtze University, Jingzhou, China; ^3^State Key Laboratory for Diagnosis and Treatment of Infectious Diseases, Collaborative Innovation Center for Diagnosis and Treatment of Infectious Diseases, The First Affiliated Hospital, School of Medicine, Zhejiang University, Hangzhou, China; ^4^Jinan Microecological Biomedicine Shandong Laboratory, Jinan, China

**Keywords:** *E. faecium*, urinary tract infection, endogenous infections, transcriptome, GWAS

## Abstract

**Introduction:**

*Enterococcus faecium* is a common pathogen responsible for urinary tract infections (UTIs) and often establishes extensive colonization within the intestinal tract. Our aim was to assess the genomic and transcriptomic differences between colonized *E. faecium* without UTI (only-colonization) and colonized *E. faecium* causing UTI (endogenous infections).

**Method:**

We investigated the correlation between fecal isolates from the same patient and UTI-causing isolates using PFGE and WGS, and classified fecal isolates into two groups: those that solely colonized and those associated with endogenous urinary tract infections. We characterized the genomes of colonization-only and endogenously infected isolates by Scoary GWAS, and the transcriptomes of the isolates at 3 h urine exposure to assess pathogen-related changes.

**Result:**

Based on PFGE and WGS, eight isolates of endogenously infected *E. faecium* and nine isolates of only-colonized *E. faecium* were characterized and carbon and nitrogen regulated metabolisms such as genes encoding the phosphotransferase (PTS) system were enriched in endogenously infected *E. faecium*. Transcriptome analysis revealed significant differences in gene expression in the PTS system, lysine synthesis, galactose metabolism and citrate import between endogenously infected and only-colonized *E. faecium* isolates, highlighting the important role of certain carbon regulatory genes in the colonization and survival of endogenously infected *E. faecium*.

**Conclusion:**

In only-colonized and endogenously infected isolates, we observed differential expression patterns of genes related to carbon metabolism and amino acids, suggesting that metabolic diversity is a strategy for isolates leading to endogenous infection.

## Highlights

*Enterococcus faecium* is a common cause of urinary tract infections. Because drug resistance is prone to establish an advantage of intestinal colonization, we isolated and performed homology analysis of intestinal colonized *E. faecium* and urinary tract infections with *E. faecium* and analyzed the advantage of *E. faecium* leading to endogenous infection using genomic and transcriptomics. We identified the carbohydrate and amino acid metabolic profiles of *E. faecium* that occur endogenously to provide an advantage for metabolic shifts. Our study provides insights for the control of clinical *E. faecium* infections.

## 1. Introduction

Urinary tract infections (UTIs) are one of the most common infections both within the community and in hospitals, which affect millions of people annually resulting in a significant burden on society (McLellan and Hunstad, 2016). In the USA, indirect and direct costs associated with UTIs total more than $2 billion annually (Foxman, 2014). Signs and symptoms of UTIs range from asymptomatic bacteriuria, urinary frequency or urinary urgency, and loin pain, to severe migrations through the urinary tract to the kidneys leading to pyelonephritis developing into bacteremia and eventually leading to death (Mody and Juthani-Mehta, 2014).

*Enterococci* are part of the intestinal microbiota and are also a common UTI causing pathogen. A decade-long study indicates that *Enterococcus faecium* is the fourth most common pathogen causing urinary tract infections and is the primary pathogen in neonatal urinary tract infections (Huang et al., 2021).

*Enterococci* colonisation of the gut confer new traits *in vivo* by horizontal transfer or mutations leading to lineage diversification, driving phenotypic changes and subsequent adaptation (Moradigaravand et al., 2017; Yang et al., 2022). Previous study have shown that colonized-*Enterococci* are reservoirs of hospital-associated infections that can easily lead to endogenous UTIs via the anus-perineum-urethra (Magruder et al., 2019). Hospital-adapted enterococcal lineages are capable of withstanding the pressure of external antibiotics and host immunity, which allows them to become the dominant pathogen in infections (Van Tyne et al., 2019). Some studies indicate that infectious *Enterococci* carry more virulence genes and biofilm genes than colonized *Enterococci* (Rigottier-Gois et al., 2015; Marchi et al., 2018). However, no single factor has proven to be necessary for *E. faecium* infection, and the adaptive genes responsible for *E. faecium* colonization of the urethra are unknown.

The intestinal tract differs significantly from the urinary tract in nutrient availability and immune clearance. To survive, *E. faecium* must respond to the change in environment from the intestines to the urinary tract. *E. faecium* utilizes different carbon sources to survive and the subsequent metabolism results in different isolate patterns in the host ecological niche (Gilmore et al., 2014). In addition, genes for *de novo* nucleotide biosynthesis and phosphotransferase system subunits contribute to the growth of *E. faecium* in serum (Zhang et al., 2017). Thus, the ability of *E. faecium* to cause UTIs may involve expression of metabolic genes in the urinary tract.

To our knowledge, the factors leading to endogenous infection with *E. faecium* have not been fully understood. Thus, we aimed to compare the genome and transcriptome of only-colonized *E. faecium* isolates (i.e., fecal isolates distinct from urinary tract infected isolates) with those of *E. faecium* isolates with endogenous infection (i.e., fecal isolates identical to urinary tract infected isolates). Our study will provide insights into endogenous infections of *E. faecium*.

## 2. Method

### 2.1. Sourcing of *E. faecium* isolates

This retrospective study was conducted at The First Hospital Affiliated to Zhejiang University School of Medicine. From January 2018 to December 2020, *E. faecium* from the urinary and intestinal tracts were screened in our LIS system from the same patients, and eligible bacteria were thawed and isolated in our bacterial bank. In our study, a total of 49 strains of *E. faecium* were included. Bacteria were stored in strain preservation solution at −80°C. We used MALDI-TOF/MS to determine the identity of the isolates. The isolates were named PX-YY-GI/U, where PX is the patient, YY is the isolate ID, GI is the gastrointestinal isolate, and U is the urinary tract infection isolate.

### 2.2. Antibacterial drug susceptibility determination

Minimum inhibitory concentrations (MIC) were determined according to drug susceptibility guidelines established by the American Clinical and Laboratory Standards Institute (CLSI). The agar dilution method or broth dilution method was used to assess MIC for penicillin, ampicillin, vancomycin, teicoplanin, erythromycin, tetracycline, ciprofloxacin, levofloxacin, rifampicin, linezolid, and tigecycline, daptomycin. *Enterococcus faecalis* ATCC29212 was used for quality control.

### 2.3. PFGE to determine homology of colonized isolates with UTI isolates

PFGE was performed to assess homology of isolated *E. faecium*, and the assay was performed as described previously with slight modifications (Erdem et al., 2020). Briefly, bacteria encapsulated in 1%v/v SDS agar and 1% SeaKem Gold agarose plugs were lysed using lysozyme and proteinase K. The bacterial genome was then cleaved with 50 U samI, and the fragmented DNA samples were loaded in 1% ultrapure agarose gels in 0.5 × TBE buffer with the following electrophoretic conditions: 3 s initial switching time, 20 s final switching time, 20 h running time, 120° angle, 6 V/cm gradient, 14°C temperature. The gels were stained with EthBr for 20 min, and gel fingerprints were observed by irradiating UV waves through the Uvitec system. Finally, a dendrogram was generated using BioNumericsV7.1 fingerprinting software to describe the relationship. Fecal isolates of patients were further classified as only-colonized (gastrointestinal isolates of patients different from the infecting UTI clone) and endogenously infected isolates (gastrointestinal -isolates similar to a urinary isolate of the same individual were defined as endogenous infection).

### 2.4. Bacterial DNA extraction and whole genome sequencing

We used the FastDNA^®^ Spin Kit for Soil purification kit (MP Biomedicals, Illkirch, France) to extract genomic DNA from all *E. faecium* isolates, purified DNA was sent to Whole-genome sequencing (WGS). WGS was performed using an Illumina HiSeq 2,500 instrument (Illumina, San Diego, CA, United States). Raw sequence reads were quality controlled using FastQC v.0.11.5 and trimmed using Trimmomatic v.0.40. Trimmed reads were assembled using SPAdes v.3.6. All assembled genome annotations were carried out using Prokka v.1.14.6. Roary v.3.12.0 was used for pan-genomic analysis of the annotated genomes, and Scoary v.1.6.16 was used to assess the association between the presence and absence of genes in UTIs. All genomes were analyzed for antibiotic resistance genes and virulence genes using Abricate v.1.0.1. Core-gene single-nucleotide polymorphisms (SNPs) were extracted by default parameters in snippy v.4.6.0. Putative duplicate regions, mobile genetic elements and recombination in the files were filtered using Gubbins v.2.3.4, an SNP-based maximum likelihood tree was inferred by FastTree v.2.1.5 and visualized using the ITOL web server. Multi-locus sequence typing (MLST) was performed using MLST v.2.22.0.

### 2.5. Urine exposure test for *E. faecium*

Bacteria were exposed to urine as described previously (Paudel et al., 2021). Briefly, Brain–Heart Infusion Broth (BHI) was inoculated with single colonies of *Enterococci* and shaken overnight at 200 rpm until the optical density (OD) at 600 nm was 0.4. After obtaining informed consent from each volunteer, urine was collected from healthy males and healthy females, and the urine was immediately filtered twice using a 0.22 μm membrane and stored at −80°C. Urine from three volunteers was then heated to 37°C and mixed, then the logarithmic growth phase *Enterococci* were exposed to urine for 3 h. After exposure, the bacteria were centrifuged, washed with PBS, and immediately preserved in liquid nitrogen.

### 2.6. Bacterial RNA extraction and RNA sequencing

We extracted bacterial RNA using the Rneasy^®^Plus Mini Kit (QIAGEN). RNA-seq was performed on a HiSeq 2,500 instrument from Meiji Biotechnology Inc. (Shanghai, China). Raw datas were quality checked using FastQC v.0.11.5, and low-quality reads were trimmed using Trimmomatic v.0.40. The filtered data were mapped to the assembled genome using Hista2 v. 2.2.1, and hit counts for encoding genes were generated using the featucounts command in Rsubread v. 2.8.1. Differential expression between control and experimental groups was detected by Deseq2, and those with adjusted *p*-values less than 0.01, Log2-fold change (Log2FC) greater than 1 were classified as differentially expressed genes (DEG). DEG was extracted for gene-ontology (GO) enrichment analysis and Kyoto Encyclopedia of Genes and Genomes (KEGG) pathway enrichment analysis.

## 3. Results

### 3.1. Patients and clinical isolates of *E. faecium*

**Figure 1:**
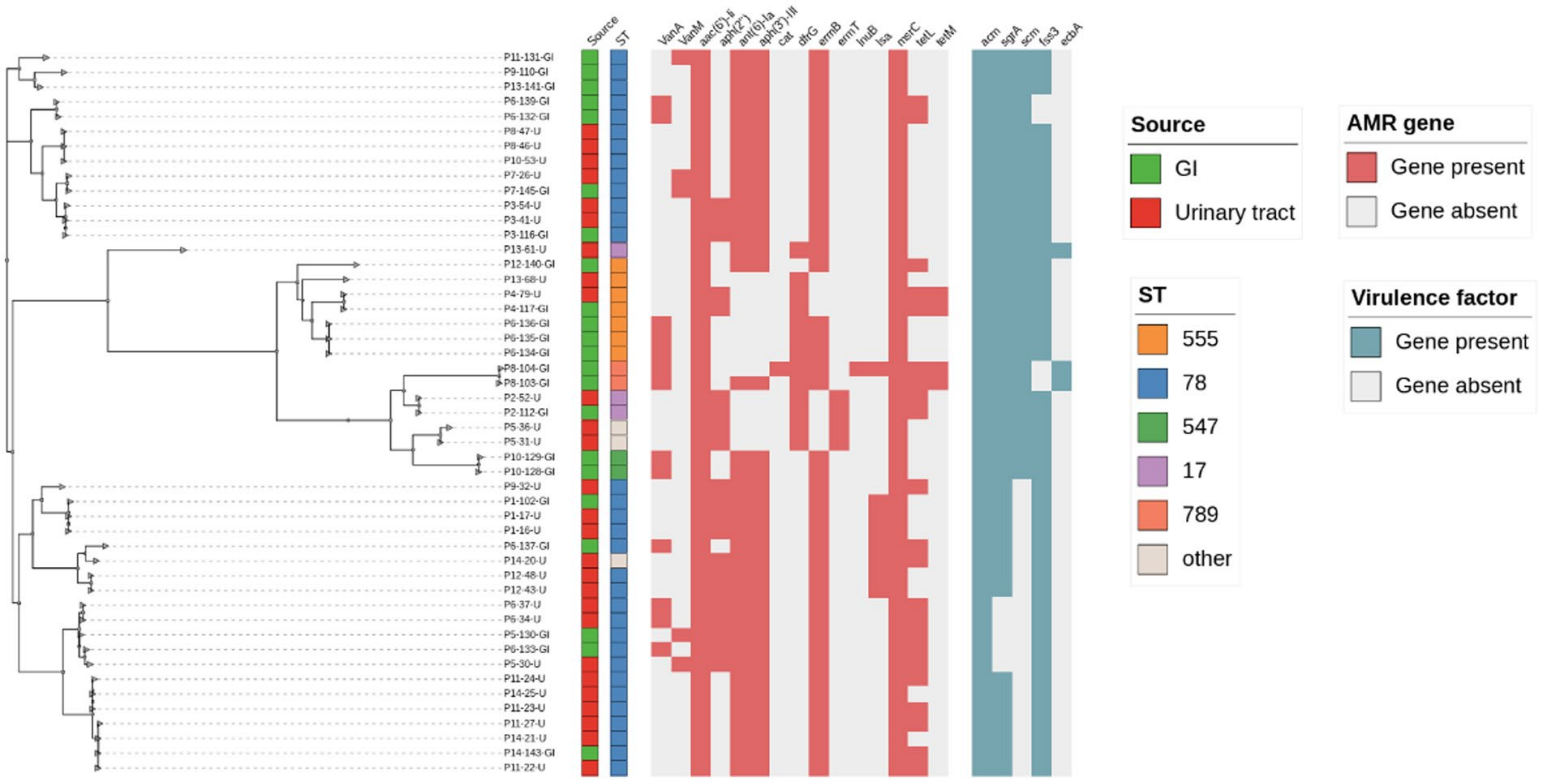
Core SNP-based phylogenetic and molecular typing, drug resistance genes, and virulence gene analysis of *E. faecium*.

Due to the possible differences in *E. faecium* species between gastrointestinal colonization and urinary tract infection (UTI) patients, we included as many *E. faecium* strains meeting the screening criteria as possible in our bacterial repository. We isolated a total of 49 *E. faecium* strains from 14 UTI patients, with 22 of them being colonization strains. In our cohort, *E. faecium* isolates belonged to the multilocus sequence typing (ST) ST78 (*n* = 32), ST = 555 (*n* = 9), which is a well-known multidrug-resistant and hospital-adapted spectrum in China, ST78 caused the infection in 11 patients. Analysis revealed that isolates from 8 infected patients were closely related genetically to previously colonized isolates, defining them as endogenous infections. Infections from the same patient had almost no single nucleotide polymorphism (SNP) differences (i.e., less than 7 SNPs per evolutionary year), and this high-resolution genomic evidence suggests that UTIs caused by *E. faecium* are induced by colonization strains from the same patient. The colonization isolates from 6 patients showed no correlation with urinary isolates (i.e., no endogenous infections). Resistance genes for aminoglycosides, tetracyclines, lincomycin, and peptides were detected in the isolates, and the infecting *E. faecium* isolates from the same patient’s colonization exhibited similar resistance genes and resistance phenotypes, as shown in Figure 1.

### 3.2. Only-colonized isolates genome compared to endogenous infected isolates

Based on PFGE and WGS, the intestinal colonized isolates were further differentiated into eight endogenously infected isolates (i.e., fecal isolates similar to urine isolates from the same individual) and nine only-colonized isolates (i.e., fecal isolates from patients with different UTI clones from the infected isolate, 5/14 fecal isolates considered as duplicate isolates were excluded; Table 1). Endogenously infected isolates and only-colonized isolates share similar resistance patterns and virulence genes.

**Table 1:** Data for the only-colonized group and endogenous infections group.

Isolate name	MLST	Group	Antibiotic resistance phenotype	Virulence gene profile
P6-132-GI	78	only-colonized	AMP, PG, ERY, CIP, LVX, RIF, VAN, TEC	acm, sgra, scm
P11-131-GI	78	only-colonized	AMP, PG, ERY, CIP, LVX, RIF	acm, sgra, scm, fss3
P9-110-GI	78	only-colonized	AMP, PG, ERY, CIP, LVX	acm, sgra, scm, fss3
P13-141-GI	78	only-colonized	AMP, PG, ERY, CIP, LVX, RIF	acm, sgra, scm, fss3
P6-137-GI	78	only-colonized	AMP, PG, ERY, CIP, LVX, RIF, VAN, TEC	acm, sgra, fss3
P12-140-GI	555	only-colonized	AMP, PG, ERY, CIP, LVX, RIF, TCY	acm, sgra, scm, fss3
P6-134-GI	555	only-colonized	AMP, PG, ERY, CIP, LVX, RIF, VAN, TEC	acm, sgra, scm, fss3
P10-128-GI	547	only-colonized	AMP, PG, ERY, CIP, LVX, RIF, VAN, TEC	acm, sgra, scm, fss3
P8-103-GI	789	only-colonized	AMP, PG, ERY, CIP, LVX, RIF, VAN, TEC, TCY	acm, sgra, scm, ecbA
P3-116-GI	78	endogenously infected	AMP, PG, ERY, CIP, LVX, RIF	acm, sgra, scm, fss3
P7-145-GI	78	endogenously infected	AMP, PG, ERY, CIP, LVX, RIF	acm, sgra, scm, fss3
P1-102-GI	78	endogenously infected	AMP, PG, ERY, CIP, LVX, RIF	acm, sgra, fss3
P14-143-GI	78	endogenously infected	AMP, PG, ERY, CIP, LVX, RIF	acm, sgra, fss3
P6-133-GI	78	endogenously infected	AMP, PG, ERY, CIP, LVX, RIF, VAN, TEC	acm, fss3
P5-130-GI	78	endogenously infected	AMP, PG, ERY, CIP, LVX, RIF	acm, fss3
P4-117-GI	555	endogenously infected	AMP, PG, CIP, LVX, RIF, TCY	acm, sgra, scm, fss3
P2-112-GI	17	endogenously infected	AMP, PG, ERY, CIP, LVX, RIF, TCY	acm, sgra, scm, fss3

Only-colonized, fecal isolates from patients with different UTI clones from the infected one; endogenously infected, fecal isolates similar to urine isolates from the same individual. AMP, ampicillin; ERY, erythromycin; CIP, ciprofloxacin; LVX, levofloxacin; RIF, rifampin; VAN, vancomycin; TEC, tetracycline; TCY, Teicoplanin.

**Figure 2:**
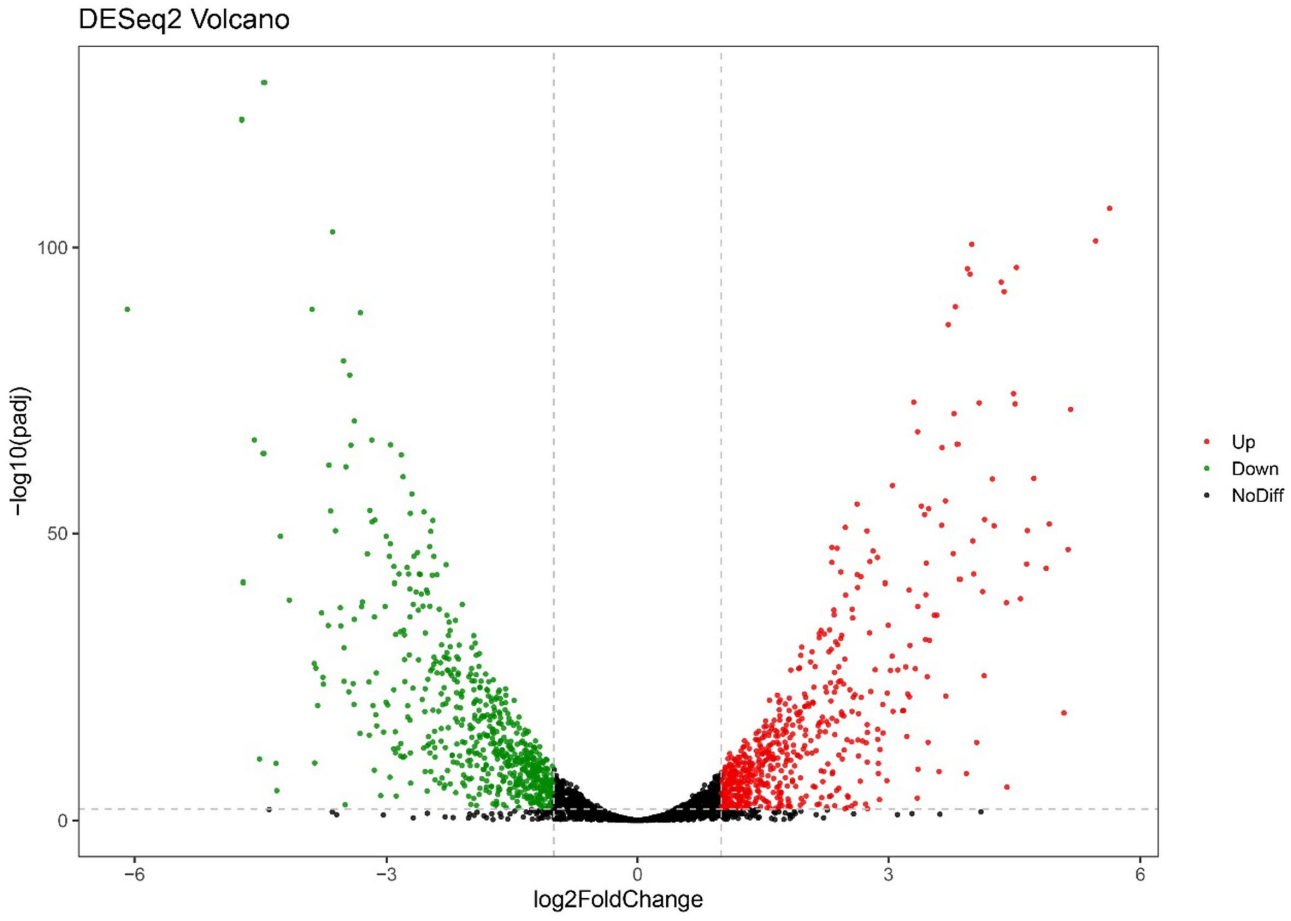
Volcano plots of RNA-seq gene counts of only-colonized *E. faecium* after exposed to urine.

*E. faecium* found in different ecological niches exhibit different genomic profiles (Kim et al., 2016), and we hypothesized that endogenously infected isolates are more adapted to the urinary environment than only-colonized isolates carrying genes specific to the intestinal environment. Therefore, we identified genes significantly associated with endogenously infected isolates by Scoary. Analysis showed that 28 genes were strongly associated with urinary pathogenicity of intestinal colonization infections based on strict *p* values (Table 2; Figure 2). Scoary showed that three genes encoding the PTS (phosphotransferase) system (manZ-6, sorA-3, manX-5) were predicted to be related to endogenous UTIs, encoding the mannose-specific EIID component, the sorbose-specific EIIC component, and the mannose-specific EIIAB component involved in substrate metabolic transport. Eight of the 23 identified endogenously infected isolate-related genes, as well as three genes encoding PTS functions were annotated while the remaining 15 were hypothetical. Eight genes were annotated for the following functions: bifunctional AAC/APH (*aacA-aphD*), ISL3 family transposase ISEfa11 (*group_2760*), L-glyceraldehyde 3-phosphate reductase (*gpr_2*), ISL3 family transposase IS1476 (*group_362*), replication initiation protein (*repN*), IS3 family transposase ISPa31 (*group_1,471*), IS3 family transposase ISSde10 (*group_1472*), and fructosamine deglycase (*frlB*). Further, we identified two genes (*rpiR* and *gltC*), encoding the helix-turn-helix (HTH)-type transcription factor, enriched in only-GI isolates, which were present in only one endogenously infected isolates. Importantly, a critical gene involved in the regulation of virulence metabolism is rpiR, while *gltC* is involved in activating expression of the glutamine-2-oxoglutarate aminotransferase gene for glutamate production. These results suggest that endogenously infected *E. faecium* isolates have a specific and nutritional/environmental adaptive gene profile, which distinguishes them from colonized isolates.

**Table 2:** Genes associated with significant differences in proportions between only-colonized and endogenously infected isolates.

Gene	Annotation	Odds_ratio	*p*_value
*group_1467*	hypothetical protein	NS	0.00905
*aacA-aphD*	Bifunctional AAC/APH	24	0.01522
*rpiR*	HTH-type transcriptional regulator RpiR	0	0.029412
*group_1075*	hypothetical protein	NS	0.029412
*group_1076*	hypothetical protein	NS	0.029412
*group_1078*	hypothetical protein	NS	0.029412
*group_2760*	ISL3 family transposase ISEfa11	NS	0.029412
*group_2761*	hypothetical protein	NS	0.029412
*group_2771*	hypothetical protein	NS	0.029412
*gpr_2*	L-glyceraldehyde 3-phosphate reductase	NS	0.029412
*group_309*	hypothetical protein	NS	0.029412
*group_362*	ISL3 family transposase IS1476	NS	0.029412
*group_5019*	hypothetical protein	NS	0.029412
*gltC_2*	HTH-type transcriptional regulator GltC	0.07	0.049774
*group_1468*	hypothetical protein	13.3	0.049774
*group_1469*	hypothetical protein	13.3	0.049774
*repN*	Replication initiation protein	13.3	0.049774
*group_1,471*	IS3 family transposase ISPa31	13.3	0.049774
*group_1472*	IS3 family transposase ISSde10	13.3	0.049774
*group_1573*	hypothetical protein	13.3	0.049774
*frlB_2*	Fructosamine deglycase FrlB	13.3	0.049774
*manZ_6*	PTS system mannose-specific EIID component	13.3	0.049774
*sorA_3*	PTS system sorbose-specific EIIC component	13.3	0.049774
*manX_5*	PTS system mannose-specific EIIAB component	13.3	0.049774
*group_2397*	hypothetical protein	13.3	0.049774
*group_635*	hypothetical protein	13.3	0.049774
*group_905*	hypothetical protein	13.3	0.049774
*group_533*	hypothetical protein	0.07	0.049774

IS, insertion sequences; NS, not significant.

### 3.3. RNA-seq reveals differential expression of endogenously infected isolates and only-colonized isolates after urine exposure

#### 3.3.1. Global transcriptional responses

One endogenously infected (p6-137-GI) isolate and one only-colonized (P6-133-GI) isolate were selected for this study. These were isolated from the same patient and both belong to ST78. Due to differences in the genomes of endogenously infected isolates and only-colonized isolates, we hypothesized that they may exhibit different transcriptional dynamics when infecting the urinary tract and that endogenously infected isolates show upregulation of genes implicated in pathogenicity. Thus, to maximize the possibility of discovering regulatory genes in UTIs, we utilized RNA-seq to compare *E. faecium* transcriptomic results between BHI growth and 3 h after urine exposure. The global transcriptomes were determined for two isolates for both conditions, and significantly DEGs were identified.

**Figure 3:**
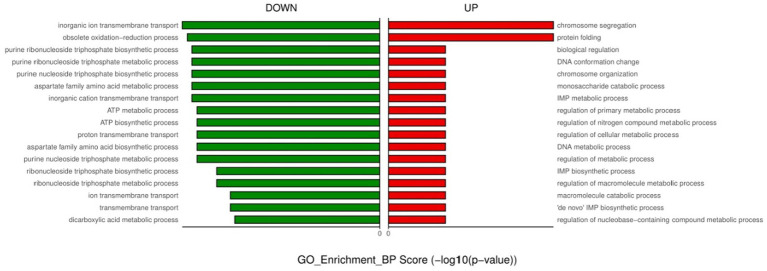
GO enrichment analysis of down- and up-regulated genes after urine exposure to only-colonized *E. faecium*.

**Figure 4:**
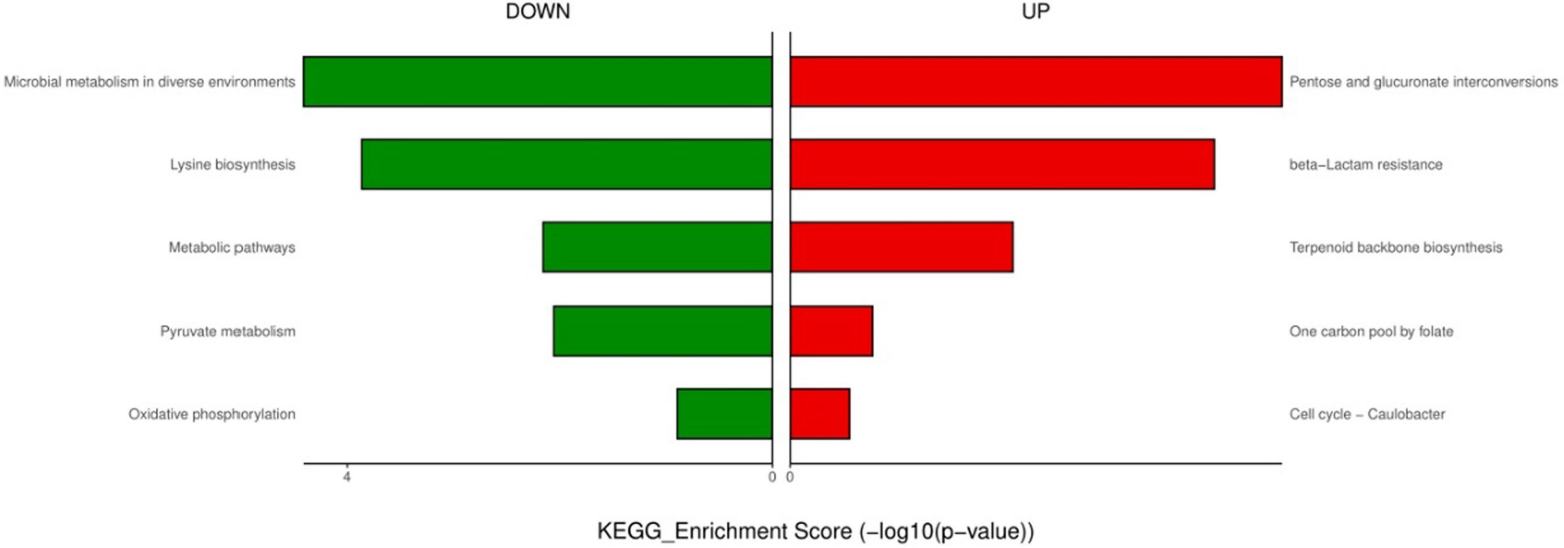
KEGG enrichment analysis of down- and up-regulated genes after urine exposure to only-colonized *E. faecium*.

For the only-colonized isolates, a total of 1,179 genes were significantly altered after urine exposure, of which 571 genes were up-regulated and 608 genes were down-regulated (Figure 2). GO enrichment analysis was performed to describe the biological processes of DEGs in more detail, and GO analysis was performed for up- and down-regulated genes, with *p* < 0.05 as the threshold of significance. Only-colonized *E. faecium* presented up-regulated expression of gene related to chromosome segregation and protein folding, as well as down-regulated expression of genes related to ion transmembrane transport, purine nucleoside metabolism, amino acid metabolism, and ATP metabolism (Figure 3). Moreover, KEGG pathway analysis was performed based on the DEGs and the implicated gene pathways were identified (Figure 4). Up-regulated gene were involved in pentose and glucuronate interconversions and beta−lactam resistance. Down-regulated genes in the KEGG pathway annotation are mainly were involved in microbial metabolism in diverse environments, Lysine biosynthesis, Metabolic pathways and Pyruvate metabolism.

**Figure 5:**
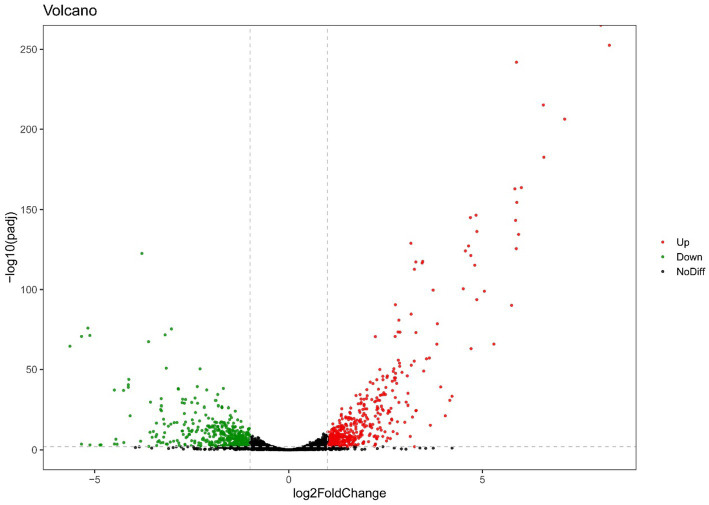
Volcano plot of RNA-seq gene counts of endogenously infected *E. faecium* after urine exposure.

**Figure 6:**
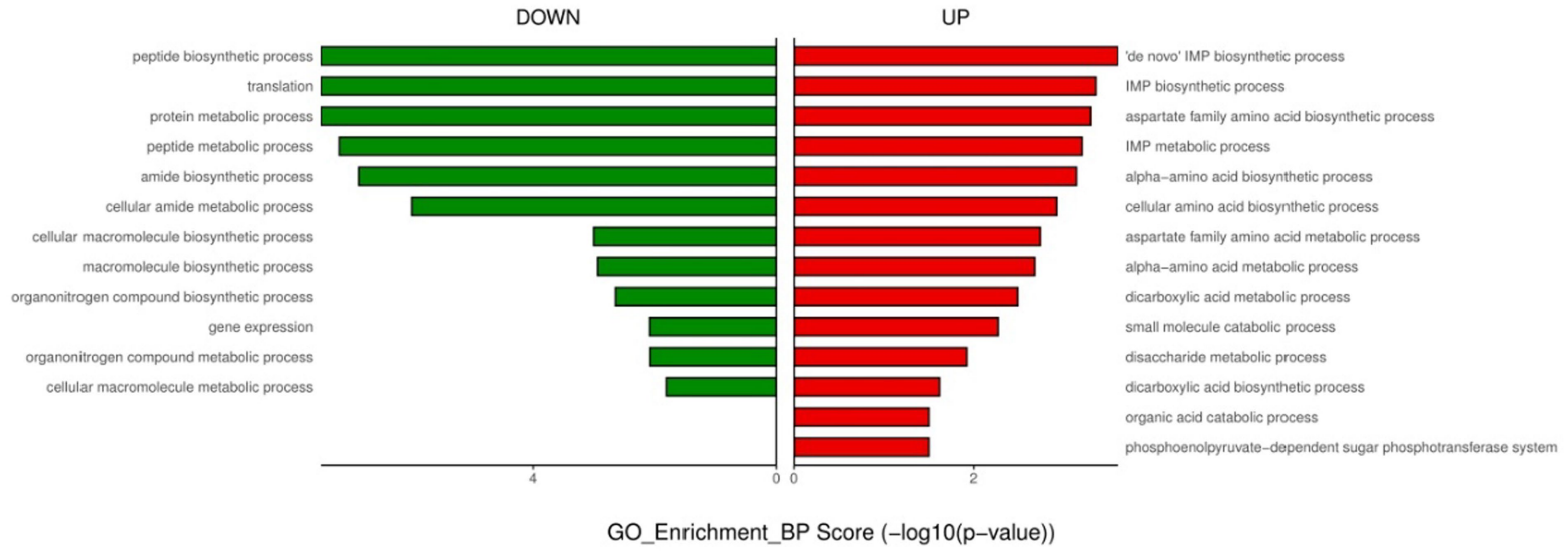
GO enrichment analysis of down- and up-regulated genes after urine exposure of endogenously infected *E. faecium*.

**Figure 7:**
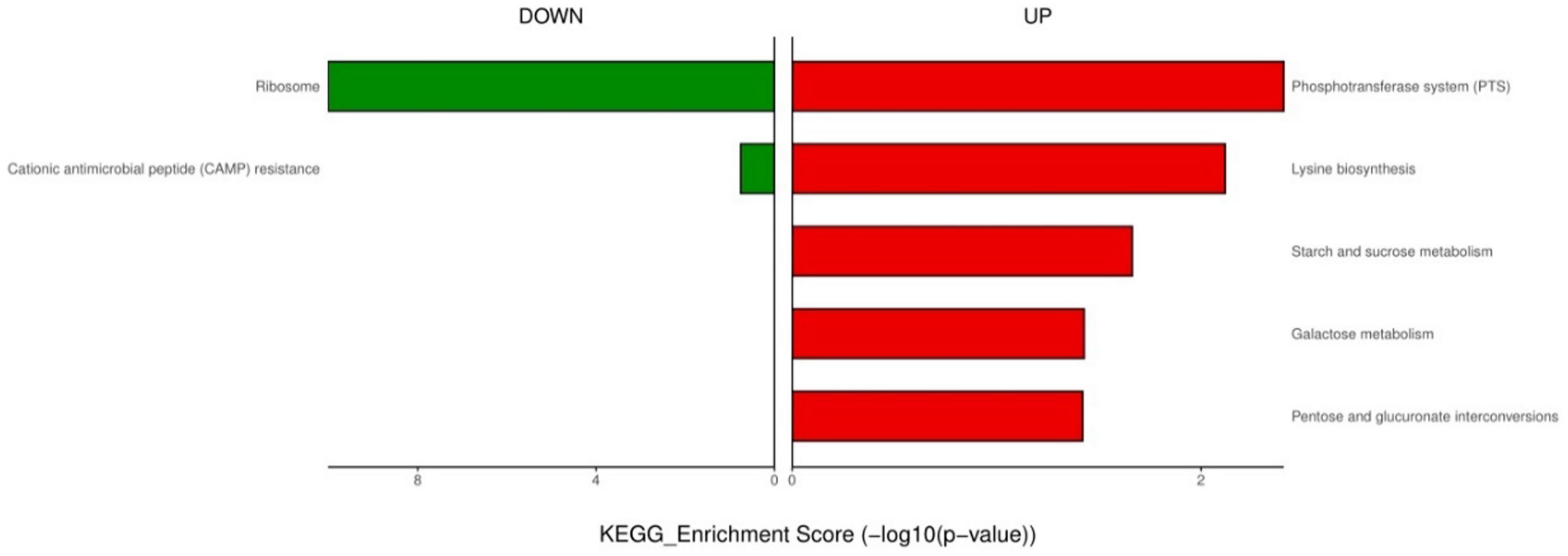
KEGG enrichment analysis of down- and up-regulated genes after urine exposure of endogenously infected *E. faecium*.

For endogenously infected isolates, urine exposure resulted in 860 DEGs in the endogenously infected isolates, of which 440 were up-regulated and 420 were down-regulated (Figure 5). GO analysis was conducted for the up- and down-regulated genes. Endogenously infected *E. faecium* exhibited upregulated expression of gene related to IMP biosynthetic and metabolic processing, amino acid biosynthetic and metabolic processing, disaccharide metabolism, dicarboxylic acid biosynthesis, organic acid catabolism as well as the phosphoenolpyruvate-dependent sugar phosphotransferase system. We also identified down-regulated expression of genes related to peptide biosynthesis and metabolism, amide biosynthesis and metabolism, organonitrogen compound biosynthesis and metabolism, as well as macromolecule biosynthesis and metabolism (Figure 6). KEGG pathway analysis showed that DEGs were most closed related to the phosphotransferase system (PTS), lysine biosynthesis, starch and sucrose metabolism, galactose metabolism and pentose and glucuronate interconversions (Figure 7).

#### 3.3.2. Biochemical pathways and genes that exhibit the same expression pattern between only-colonized isolates and endogenously infected isolates

The RNA-seq results demonstrate that only the isolates colonized and endogenously infected exhibit upregulation of identical genes after exposure to urine (Table 3). Following a 3 h exposure to glucose-free urine, the *ccpB* gene, which mediates metabolic inhibition, was upregulated in both *E. faecium* isolates to switch substrate utilization patterns to adapt to the urine environment. In addition, the *oppB* gene, encoding an oligopeptide transporter systemic permease protein, and the *oppDF* gene, an oligopeptide transporter ATP-binding protein, were up-regulated, facilitating the capture of peptides as a source of carbon and nitrogen in the bacterial cytoplasm by the strain in the extra-bacterial environment. Although urine is an unfavorable environment for *Enterococci*, no distinction was made to the stress transcription patterns of endogenously infected isolates versus only-colonized isolates. For example, *ssaB*, encoding a manganese ABC transporter substrate binding lipoprotein, *mntB*, a membrane protein of the manganese transporter system, and *scaC*, a manganese import ATP binding protein, were up-regulated in both isolates. Similarly, we observed that the most significant upregulation in response to extracellular *Cu* ions in the only-colonized versus endogenously infected isolates was the *copABZ* gene cluster, which encodes the putative copper-exporting P-type ATPase A (copA), copper-exporting P-type ATPase B(*copB*), and copper chaperone (*copZ*). We observed the regulation of cellular Cu homeostasis by both isolates, thereby up-regulating the interaction of cytoplasmic copper chaperone and Cu-ATPase to deliver more copper into the ion permeation path. In addition, *pur* genes encode enzymes that are essential in the *ab initio* synthesis of purines, and all bacteria require purine nucleotides to complete essential metabolic processes such as DNA, RNA, and protein synthesis, and similar upregulated expression was observed in both isolates.

**Table 3:** Endogenously infected *E. faecium* show similar differential expression patterns in specific biochemical pathways or genes when changed from BHI to urine growth conditions as only-colonized *E. faecium*.

Gene	Gene annotation	Fecal-UTI log2FC	Only-Fecal log2FC
Carbon catabolism regulation			
*ccpB*	Catabolite control protein B	8.04	4.52
*Oligopeptide transport*			
*oppB*	Oligopeptide transport system permease protein OppB	3.08	3.22
*oppD_1*	Oligopeptide transport ATP-binding protein OppD	1.68	NS
*oppD_2*	Oligopeptide transport ATP-binding protein OppD	3.82	3.54
*oppF_1*	Oligopeptide transport ATP-binding protein OppF	1.85	NS
*oppF_2*	Oligopeptide transport ATP-binding protein OppF	3.48	3.49
*Ion transport*			
*ssaB_1*	Manganese ABC transporter substrate-binding lipoprotein	1.12	5.14
*ssaB_2*	Manganese ABC transporter substrate-binding lipoprotein	2.84	2.99
*mntB_1*	Manganese transport system membrane protein MntB	NS	4.40
*mntB_2*	Manganese transport system membrane protein MntB	2.61	2.69
*scaC_1*	Manganese import ATP-binding protein ScaC	−1.07	3.46
*scaC_2*	Manganese import ATP-binding protein ScaC	1.89	2.56
*copA*	Copper-exporting P-type ATPase A	1.47	3.04
*copB*	Copper-exporting P-type ATPase B	NS	2.64
*copZ*	Copper chaperone CopZ	1.31	2.26
*Purine de novo synthesis*			
*purM*	Phosphoribosylformylglycinamidine cyclo-ligase	4.21	2.87
*purD*	Phosphoribosylamine--glycine ligase	4.16	2.78
*purN*	Phosphoribosylglycinamide formyltransferase	4.04	2.88
*purH*	Bifunctional purine biosynthesis protein PurH	3.92	3.11
*purS*	Phosphoribosylformylglycinamidine synthase subunit PurS	3.25	NS
*purL*	Phosphoribosylformylglycinamidine synthase subunit PurL	2.79	2.90
*purE*	N5-carboxyaminoimidazole ribonucleotide mutase	2.27	2.98
*Pentose and glucuronate interconversions*			
*uxuA*	Mannonate dehydratase	1.01	4.87
*por*	Polyol:NADP oxidoreductase	1.44	4.64
*sgbE*	L-ribulose-5-phosphate 4-epimerase SgbE	2.87	4.14
*araA*	L-arabinose isomerase	3.24	3.93
*uxaC*	Uronate isomerase	3.28	3.82
*xylB_2*	Xylulose kinase	2.54	3.68
*uxaA*	Altronate dehydratase	2.51	3.64
*uxaB*	Altronate oxidoreductase	3.56	3.43

ABC, ATP-binding cassette; NADP, nicotinamide adenine dinucleotide phosphate.

#### 3.3.3. Biochemical pathways and genes exhibiting different expression patterns between only-colonized isolates and endogenously infected isolates

Although *E. faecium* isolates showed similar differences when transferred from the BHI to the urine, there were differences in carbon source metabolism and amino acid metabolism between only-colonized and endogenously infected isolates (Table 4).

**Table 4:** Endogenously infected *E. faecium* show differential expression in specific metabolism or gene expression compared to only-colonized *E. faecium* with BHI conversion to urine.

Gene	Gene annotation	Fecal-UTI log2FC	Only-Fecal log2FC
Lysine synthesis			
*dapA*	4-hydroxy-tetrahydrodipicolinate synthase	2.39	−1.46
*dapB*	4-hydroxy-tetrahydrodipicolinate reductase	2.69	−1.38
*dapF*	Diaminopimelate epimerase	2.27	−1.53
*dapH*	2,3,4,5-tetrahydropyridine-2,6-dicarboxylate N-acetyltransferase	3.19	−1.36
*dapX*	putative N-acetyl-LL-diaminopimelate aminotransferase	1.57	−1.34
*lysA*	Diaminopimelate decarboxylase	1.49	−1.20
*yclM*	Aspartokinase 3	2.37	−1.60
*asD*	Aspartate-semialdehyde dehydrogenase	2.66	−1.15
*Galactose metabolism*			
*lacA*	Galactose-6-phosphate isomerase subunit LacA	4.70	
*lacB*	Galactose-6-phosphate isomerase subunit LacB	4.69	
*lacC_2*	Tagatose-6-phosphate kinase	4.84	
*lacD_1*	Tagatose 1,6-diphosphate aldolasec	4.86	
*lacD2*	Tagatose 1,6-diphosphate aldolase 2	4.49	
*lacE_1*	PTS system lactose-specific EIICB component	2.71	
*lacE_3*	PTS system lactose-specific EIICB component	4.64	
*lacF_1*	PTS system lactose-specific EIIA component	2.35	
*lacF_5*	PTS system lactose-specific EIIA component	4.56	
*lacG_1*	6-phospho-beta-galactosidase	1.97	
*lacL*	Beta-galactosidase large subunit		2.41
*gatc_3*	PTS system galactitol-specific EIIC component	1.39	−4.7
*galM*	Aldose 1-epimerase	1.38	2.74
*galK_2*	Galactokinase	1.24	
*gatA_2*	PTS system galactitol-specific EIIA component	1.11	
*galE*	UDP-glucose 4-epimerase		2.46
*galT*	Galactose-1-phosphate uridylyltransferase		2.40
*uidA_2*	Beta-glucuronidase		3.23
*Citrate synthesis*			
*citC*	[Citrate [pro-3S]-lyase] ligase	6.59	
*citD*	Citrate lyase acyl carrier protein	6.58	
*cimH*	Citrate/malate transporter	6.00	
*citE*	Citrate lyase subunit beta	5.89	
*citX*	Apo-citrate lyase phosphoribosyl-dephospho-CoA transferase	5.88	
*citF*	Citrate lyase alpha chain	5.86	
*citG*	2-(5″-triphosphoribosyl)-3′-dephosphocoenzyme-A synthase	5.84	

In endogenously infected isolates, the *yclM* and *asd* operons, which are responsible for the conversion of aspartate to aspartate semialdehyde, were up-regulated after urine exposure. However, the *yclM* and *asd* operons were down-regulated in the only-colonized isolates. Aspartate semialdehyde is metabolized to synthesize lysine, and the gene cluster (*dap* operon) involved in the formation of lysine precursors from aspartate semialdehyde via the diaminopyrimidine (DAP) pathway showed consistent up-regulation in endogenously infected isolates, However the lysine biosynthetic pathway was significantly down-regulated in the only-GI isolates after urine exposure. Further, marked differences in the metabolic lactose pathway were observed between the endogenously infected isolates and the only-colonized isolates. The *lacEF* gene was significantly up- and down-regulated in the urine-exposed endogenously infected isolates and in the only-colonized isolates, respectively. *LacEF* encodes for phosphoenolpyruvate (PEP)-dependent phosphotransferase, which transfers lactose to the extracellular bacterial environment and produces lactose-6-phosphate. Similarly, *LacG*, which encodes a phosphorylated-β-galactosidase that hydrolyzes to produce lactose-6-phosphate and glucose, was up-regulated in urine-exposed endogenously infected isolates. Further, compared to BHI, endogenously infected isolates also up-regulated the tagatose-6-phosphate pathway-related operons (*lacABCD*) responsible for galactose-6-phosphate catabolism following urine exposure. Moreover, only-colonized isolates up-regulated the Leloir pathway-related operons (*galK*, *galE*, and *galT*). In addition, *cit*-encoding genes that mediate citrate transport and utilization were significantly expressed in endogenously infected isolates.

## 4. Discussion

*E. faecium* is a significant causative pathogen for healthcare-associated infections, colonizing the intestines and easily infecting the urinary tract. Here, we describe comparative genomics and transcriptomics to identify and characterize specific genetic factors and adaptations of *E. faecium* from enteric colonization and UTI pathogenic colonization. Our findings highlight that (McLellan and Hunstad, 2016) *E. faecium* isolates colonized in the intestine are capable of causing UTIs and (Foxman, 2014) the metabolic diversity of endogenously-infected *E. faecium* would be advantageous in a changing environment compared to only-colonized *E. faecium* and may allow colonization of the urinary tract by *E. faecium* with unique nitrogen and carbon utilization patterns. The species specificity and environmental sensitivity of *E. faecium* are major contributors to urinary tract colonization and infection. In addition, a solid understanding of the characteristics of hospital colonized *E. faecium* is essential for preventing healthcare-acquired infections.

Urine is a nutrient-poor medium, primarily composed of urea, inorganic salts, creatinine, organic acids, small amounts of amino acids, and other water-soluble waste products (Mann et al., 2017). For pathogens translocated into the urinary tract, the environmental stress in urine may damage cell physiology, potentially leading to reduced growth rates or bacterial lysis (van der Waal et al., 2012), and these stresses include osmotic pressure, nutrient depletion, or pH, among others (Naicker et al., 2020). Urine is nutritionally less supportive for bacterial growth compared to the intestinal environment; therefore, metabolic flexibility and adaptability are crucial for the survival of *E. faecium* in the urinary tract. In this context, *E. faecium* exhibits various metabolic adaptations in central carbon metabolism, amino acid metabolism, and other pathways such as purine metabolism, triggered by exposure to urine. Metabolic pathways represent a diverse category of differentially expressed genes (DEGs) according to transcriptomic results upon exposure to urine. Through GO and KEGG analyses, DEGs are generally classified into expected categories transitioning from nutrient-rich to nutrient-poor living environments. The phosphotransferase system (PTS) regulatory system includes a large gene network associated with many metabolic processes and adaptive responses, including lactose/melibiose and mannitol PTS systems. PTS is one way bacteria use for sugar uptake, where the energy source is phosphoenolpyruvate. It is involved in the transport of various sugars into bacteria, including glucose, mannitol, fructose, and disaccharides, and its activation in different environments may vary, reflecting the appropriate carbon sources available in the environment. Furthermore, extensive research has shown that PTS regulation is associated with more complex genes that affect bacterial pathogenic mechanisms, including virulence and bacterial chemotaxis towards host cells (Kok et al., 2003; Somavanshi et al., 2016; Jeckelmann and Erni, 2020).

Carbon Catabolite Repression (CCR) leads to the transcriptional inhibition of enzymes or genes involved in the utilization of other substrates when a nutrient source (such as glucose) is used (Opsata et al., 2010). However, glucose levels in urine are far below the threshold for CCR (carbon catabolite repression). We observed a significant upregulation of the *ccpB* gene, which mediates the *E. faecium* catabolic metabolism system, after exposure to urine. This led to bacteria beginning to utilize less-preferred substrates (Tables 3, 4). Our data reflect that when transitioning from a nutrient-rich BHI culture to nutrient-poor urine, bacteria quickly adapt to the necessity of coping with nutritional changes.

The concentration of citrates in human urine typically ranges from 1–2 mM (Wishart et al., 2009) and can serve as the sole carbon and energy source for *E. faecium*. In our study, genes encoding citrate transport and metabolism were upregulated in *E. faecium* after exposure to urine. This was primarily achieved through membrane transport, followed by citrate lyase cleaving citrate into acetyl coenzyme A and oxaloacetate, which is then decarboxylated into pyruvic acid, sustaining the survival of *E. faecium*. Previous research has shown that the utilization of citrates promotes biofilm formation in *Staphylococcus aureus* (Shanks et al., 2008), and there is also evidence that the loss of citrate metabolism affects the virulence expression of Enterococcus (Martino et al., 2018). When transitioning from the intestinal environment to the urine environment, the regulation of citrate uptake and catabolism by *E. faecium* may contribute to the establishment of colonization after intestinal translocation to urine and ultimately provide an advantage in infecting patients.

In this work, we found that endogenously infected *E. faecium* carries carbon-regulated metabolic genes and are more adapted to environmental changes. RNA-seq was used to further characterize transcriptional differences between endogenously infected *E. faecium* and only-colonized *E. faecium* and to show that multiple pathways involve different sets of genes that may lead to successful infection. Endogenously infected *E. faecium* was similar to only-colonized *E. faecium* in purine metabolism and oxidative stress-related pathways, and endogenously infected *E. faecium* was critical for the growth of genes related to lactose and citrate metabolism and lysine synthesis in the urine. Our results are expected to be a target for the treatment and prevention of *E. faecium* endogenous infection in nosocomial patients.

## Data availability statement

The datasets presented in this study can be found in online repositories. The names of the repository/repositories and accession number(s) can be found in the article/supplementary material.

## Author contributions

GH: Writing – original draft. YZ: Writing – review & editing. HC: Data curation, Methodology, Writing – review & editing. TL: Methodology, Formal analysis, Writing – review & editing. LZ: Resources, Software, Project administration, Writing – review & editing. CL: Resources, Visualization, Data curation, Writing – review & editing. YC: Funding acquisition, Supervision, Validation, Writing – review & editing.
